# Safety and Preliminary Clinical Effects of a Herbal Balance Solution in Mild‐To‐Moderate Acne Vulgaris: An Open‐Label, Single‐Arm Pilot Study

**DOI:** 10.1111/jocd.70702

**Published:** 2026-02-06

**Authors:** Xiaoyue Teng, Xiaohong Shu, Wei Huo, Zhaoxia Li, Lin Zou, Li Li, Xi Wang

**Affiliations:** ^1^ Department of Dermatology West China Hospital, Sichuan University Chengdu China; ^2^ Center of Cosmetic Evaluation West China Hospital, Sichuan University Chengdu China

**Keywords:** acne vulgaris, herbal balance solution, lesions, papules

## Abstract

**Objective:**

Acne is a common skin condition that seriously affects the physical and mental health of patients. In addition to the standard acne treatment, anti‐acne skin care products are gradually becoming essential in preventing and treating acne. Herbal Balance Solution contains multiple botanical ingredients with potential anti‐inflammatory and barrier‐supportive properties. This pilot study aimed to explore the safety and preliminary clinical effects of Herbal Balance Solution in patients with mild‐to‐moderate acne vulgaris.

**Methods:**

Thirty‐three patients with mild or moderate acne vulgaris were treated with Herbal Balance Solution once every 3 days for 4 weeks. At baseline (Week 0, W0), Week 2 (W2), and Week 4 (W4), acne lesion counts (total, inflammatory, and non‐inflammatory lesions) and the severity index were measured to assess the treatment efficacy, and transepidermal water loss was tested to assess face irritation.

**Results:**

The number of closed comedones was significantly reduced (*p* < 0.01) at each time point after using the test product. Furthermore, the transepidermal water loss rate significantly decreased (*p* < 0.05). At Week 4, reductions in total inflammatory and non‐inflammatory lesion counts were observed and were statistically significant (*p* < 0.05).

**Conclusion:**

In this open‐label pilot study, use of Herbal Balance Solution was associated with short‐term improvements in acne‐related clinical parameters and skin barrier function and was well tolerated in patients with mild‐to‐moderate acne vulgaris. These preliminary findings warrant confirmation in randomized, vehicle‐controlled clinical trials.

## 1 Introduction

Acne vulgaris is a chronic inflammatory skin disease of the follicular sebaceous unit that occurs predominantly during adolescence and primarily affects the face. In addition to standard acne treatment, anti‐acne skin care products are increasingly recognized as important adjunctive measures in preventing and treating acne [1]. Acne vulgaris is mainly characterized by open or closed comedones (blackheads and whiteheads) and inflammatory lesions, including papules, pustules, and nodules (cysts), associated with various genetic and environmental factors [2]. Acne incidence in the Chinese population is approximately 8.1% [3], and studies have shown that it is as high as 93% in adolescents [4]. Acne is the eighth most common chronic disease worldwide. According to statistics, 31.8% of acne vulgaris cases can be secondary to sensitive skin, and 3%–7% can leave scars, which seriously affects the self‐image and mental health of patients [5].

Currently, the standard therapies for acne vulgaris treatment primarily include topical treatment and systemic pharmacotherapy, specifically involving the use of retinoids and antibiotics. Topical treatments directly target the lesion area, reducing the risk of systemic adverse reactions and facilitating self‐administration. These treatments are especially suitable for mild to moderate acne and can also serve as an adjunct to systemic therapies for severe acne. However, topical therapies may cause skin dryness, irritative reactions, or allergic responses [2]. Retinoids have demonstrated significant efficacy in the treatment of moderate to severe acne, reducing the severity of acne lesions and preventing scar formation. Nevertheless, their use may also cause discomfort such as skin dryness, desquamation, and pruritus. In addition, owing to their potential teratogenicity, retinoids are strictly contraindicated in pregnant women [6]. Systemic antibiotic therapy can rapidly alleviate the progression of acne symptoms and is a reasonable option for patients with moderate acne. However, prolonged or improper use can lead to antibiotic resistance and adverse effects such as gastrointestinal discomfort [7]. Given these challenges, exploring a safe, efficient, easy‐to‐use, and low side effect acne treatment regimen has become an urgent research priority. Therefore, alternative therapeutic strategies with favorable safety profiles are needed. Treatment concepts and methods are constantly updated following the increased understanding of acne pathogenesis. The updated 2024 American Guidelines for the Clinical Management of Acne state that herbal and alternative therapies have been used to treat acne [8]. Although data on the safety and efficacy of these therapies remain limited, existing studies have shown that most of these products are well tolerated. Against this background, this study aimed to explore the safety and preliminary clinical effects of Herbal Balance solution—a compound preparation with nine herbal extracts as the main components, including *Houttuynia cordata, licorice root powder*, and *birch bark extract*—in individuals with acne through a prospective self‐controlled clinical study, thereby providing additional data on the use of herbal therapy in acne management.

## 2 Materials and Methods

A prospective, open‐label, single‐arm clinical study was conducted at the Cosmetics Center, West China Hospital, Sichuan University, from August to September 2023. The study protocol was approved by the Ethics Committee in 2023 (2023年审(53) 号). Informed consent was obtained from all the participants.

### 2.1 Participants

A total of 37 patients were recruited. Male and female patients aged 18–35 years with acne vulgaris of grade 2–3, according to the Global Acne Severity (GEA) scale, were included in this study [9]. Exclusion criteria included pregnancy, lactation, and age < 18 years. Participants with a lactic acid stinging test score ≥ 3, vulnerable skin diseases, sensitivity to cosmetics, or a history of allergy to any of the ingredients in the product, history of applying any topical agents within 4 weeks to the studied area, or ongoing use of drugs associated with acne treatment were excluded from the study. Patients were not permitted to receive any other systemic or topical treatment for acne vulgaris concomitantly from 1 month before treatment initiation to the completion of the study.

### 2.2 Test Substances

The Herbal Balance Solution constitutes a complex mixture, with its principal components delineated in Table 1.

**TABLE 1:** List of the primary components.

Item number	Botanical ingredient	Extract/Powder source	w/%
1	*Betula alba*	Bark extract	0.65–2.50
2	*Camellia sinensis*	Leaf powder	0.65–1.50
3	*Saposhnikovia divaricata*	Root extract	0.65–1.50
4	*Glycyrrhiza glabra*	Root powder	0.65–1.50
5	*Prunus armeniaca*	Seed powder	0.65–2.00
6	*Houttuynia cordata*	Powder	0.65–1.50
7	*Taraxacum officinale*	Leaf extract	0.65–1.50
8	*Citrus unshiu*	Fruit peel extract	0.65–2.50
9	*Phaseolus radiatus*	Seed powder	0.65–1.50
10	Lactic acid	—	0–3.00

### 2.3 Study Procedures

This study used a 4‐week, one‐group pretest–post‐test design to compare the safety and efficacy of the Herbal Balance Solution for treating acne vulgaris. After cleaning their faces, the participants were instructed to apply an appropriate amount of the Herbal Balance Solution to their faces and rinse it off with clean water after 5–15 min. This process was repeated once every 3 days throughout the study. During their first visit, data on the participants' baseline characteristics, including age, sex, and Fitzpatrick skin phototype, were collected. At each visit, a clinical assessment of the participants' facial skin condition was conducted, along with non‐invasive instrumental testing. Follow‐up visits were conducted at W0, W2, and W4. During each follow‐up visit, the participants were asked if they experienced any adverse reactions, such as burning, itching, and tingling. All adverse reactions were recorded. Testing is conducted under constant temperature and humidity conditions, with a temperature range of 20°C–22°C and a relative humidity range of 40%–60% RH. During the trial, participants were provided with usage logs to record the timing and frequency of product use. At each follow‐up visit, investigators reviewed the logs and weighed the returned study product to assess compliance with the prescribed dosage and usage instructions.

### 2.4 Evaluation Method

Lesion counts (open comedones, closed comedones, and papules) were determined through standardized in‐person facial examinations. Global acne severity was assessed using the GEA scale based on assessor‐blinded evaluation of standardized facial photographs. Pore appearance and skin sebum levels were evaluated using predefined 0–5 and 0–9 grading scales, respectively, under standardized assessment conditions. CBS‐2021 multi‐spectral MindScan imaging system (Wuhan Bose Electronic Co. Ltd., Wuhan, China), equipped with a Canon special imaging system, was used to capture images from the front and sides at 45°. This system includes a 4 K ultra‐high‐definition camera with 24 million pixels, providing high‐resolution images for detailed skin analysis. The transepidermal water loss (TEWL) was measured using the Tewameter TM300 (MPA 10, Courage + Khazaka electronic GmbH, Cologne, Germany). Regional variations in the barrier function of the stratum corneum (SC) exist across different areas of the face. The skin around the oral cavity demonstrates the poorest barrier function, as evidenced by higher TEWL values [10]. Consequently, measuring TEWL in the perioral region is particularly effective for detecting changes in water loss, as it reflects alterations in skin barrier function. Compared with other areas, the perioral region is more suitable for TEWL measurement owing to its relatively flat surface and minimal hair interference, allowing for accurate instrument placement and stable readings. The Corneometer CM825 (MPA 10, Courage + Khazaka electronic GmbH, Cologne, Germany) was used to measure the hydration of the stratum corneum (SC), and the Sebumeter SM810 (MPA 10, Courage + Khazaka electronic GmbH, Cologne, Germany) was employed to test the sebum content. TEWL measurements were repeated in the perioral area of each participant. Three tests for SC hydration were conducted on the cheekbones. Sebum content was tested once on each side of the forehead. The average of these values was calculated for analysis.

### 2.5 Statistical Analysis

All statistical analyses were performed using SPSS software (version 29.0; Inc., Chicago, IL, USA), and statistical significance was set at a *p* value of < 0.05. For patient pre‐ vs. post‐intervention comparisons, quantitative data were analyzed with the paired sample *t*‐test or the Wilcoxon signed‐rank test, depending on whether the normality assumption was met. The Wilcoxon signed‐rank test was used for ordinal data. As this was an exploratory study, a priori sample size estimation was not performed. A post hoc power analysis was conducted for the primary efficacy endpoint based on the observed effect sizes derived from the Wilcoxon signed‐rank tests.

## 3 Results

### 3.1 Demographics and Baseline Characteristics

Thirty‐seven patients with facial acne vulgaris of the face were enrolled in the study, of whom 33 completed treatment with herbal balancing liquid. Four patients were lost to follow‐up due to personal reasons. The mean age of the participants who completed the study was 24.6 ± 3.8 years (range: 18–32 years), including 5 males (15.15%) and 28 females (84.85%). The Fitzpatrick skin phototype distribution of the patients was as follows: type II, 2 (6.06%); type III, 19 (57.58%); type IV, 12 (36.36%).

### 3.2 Efficacy Evaluation

**FIGURE 1:**
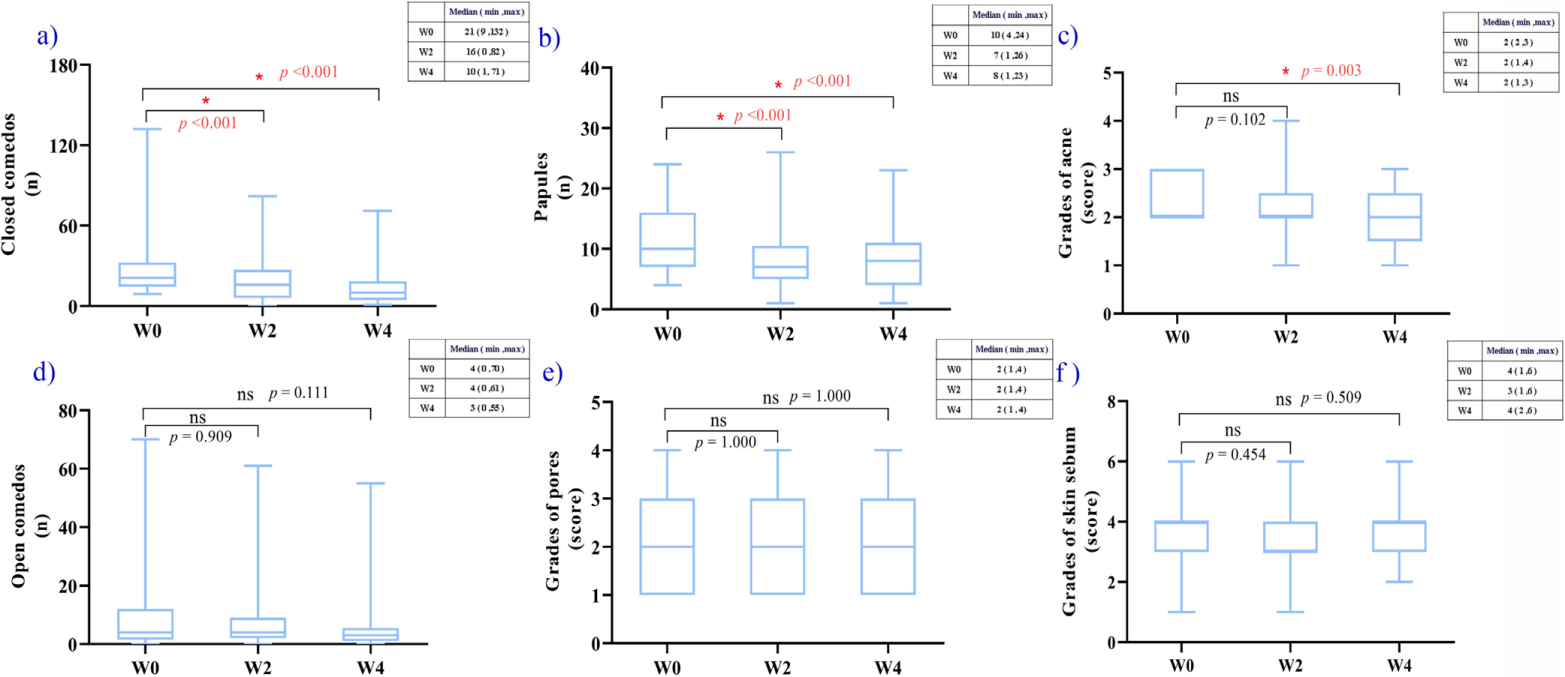
Clinical assessments at W0, W2, and W4. (a) Number of closed comedones. A statistically significant decrease versus baseline was observed after W2 and W4 for closed comedones count (*p* < 0.05). (b) Number of papules. A statistically significant decrease versus baseline was observed after W2 and W4 for papules count (*p* < 0.05). (c) Acne assessment using the investigator Global Evaluation of Acne scale. A statistically significant decrease versus baseline was observed after W4 for acne grades (*p* < 0.05). (d) Number of open comedones. (e) Grades of pores. (f) Sebum content. W0, baseline; W2, Week 2; W4, Week 4.

Based on the results of the clinical assessment, closed comedones (Figure 1a), papules (Figure 1b), and acne scores (Figure 1c) showed more significant improvements after 4 weeks. The number of closed comedones and papules showed an overall decreasing trend after 4 weeks (*p* < 0.05). Initially, the mean number of closed comedones was 30.7 ± 27.4, reducing to 20.1 ± 18.3 after 2 weeks (Wilcoxon signed‐rank test—*p* < 0.001) and 13.7 ± 14.1 after 4 weeks (Wilcoxon signed‐rank test—*p* < 0.001). Based on the observed effect sizes, the post hoc power analysis indicated that the statistical power for the primary endpoint exceeded 0.99. Prior to application, the mean number of papules was 11.8 ± 5.4, which decreased to 8.0 ± 4.9 after 2 weeks (Wilcoxon signed‐rank test—*p* < 0.001) and 8.0 ± 4.9 after 4 weeks (Paired sample *t*‐test—*p* < 0.001). When the 4 weeks results were compared with the baseline values, no significant differences were detected in any open comedones (Paired sample *t*‐test—*p* = 0.909 at 2 weeks; Paired sample *t*‐test—*p* = 0.111 at 4 weeks) or pore scores (Wilcoxon signed‐rank test—*p* = 1.000 at 2 weeks; Wilcoxon signed‐rank test—*p* = 1.000 at 4 weeks) (Figure 1d,e) (*p* > 0.05). The acne grading scores significantly improved at 4 weeks, averaging 2.3 ± 0.4 at baseline, 2.2 ± 0.7 at 2 weeks (Wilcoxon signed‐rank test—*p* = 0.102), and 2.0 ± 0.7 at 4 weeks (Wilcoxon signed‐rank test—*p* = 0.003) (Figure 1c).

**FIGURE 2:**
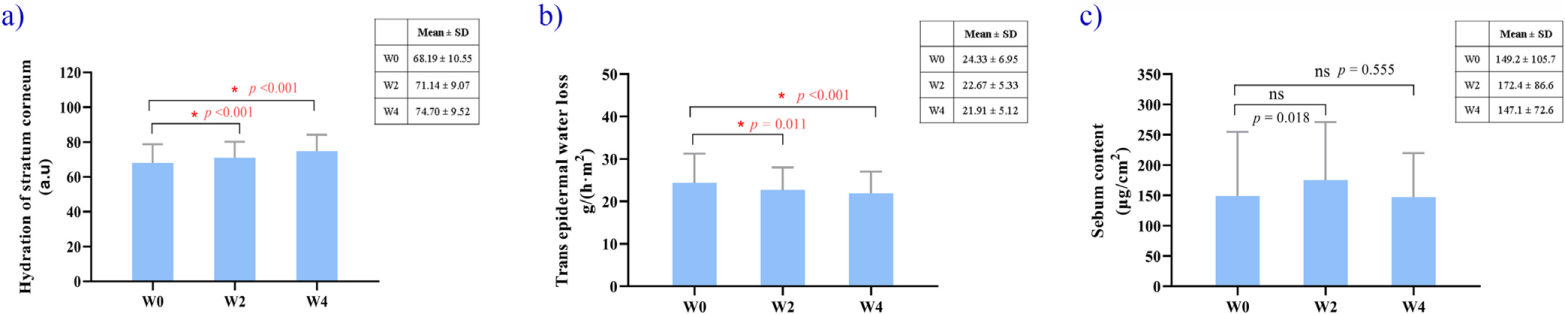
Instrumental assessments at W0, W2, and W4. Herbal Balance Solution significantly (*p* < 0.05) improved skin hydration and transepidermal water loss at W2 and W4 compared with W0. (a) Skin hydration. (b) Transepidermal water loss. (c) Sebum content. W0, baseline; W2, Week 2; W4, Week 4.

Results from the non‐invasive measurement devices, as shown in Figure 2, revealed a significant increase in skin hydration after 2 weeks (71.14 ± 9.07; Paired sample *t*‐test—*p* < 0.001) and 4 weeks (74.70 ± 9.52; Paired sample *t*‐test—*p* < 0.001) compared with baseline (68.19 ± 10.55) (Figure 2a). The Transepidermal Water Loss (TEWL) value decreased from 24.33 ± 6.95 at baseline to 22.67 ± 5.33 at 2 weeks, and further to 21.91 ± 5.12 at 4 weeks. Statistically significant reductions in TEWL values were observed at both the 2‐week (Paired sample *t*‐test—*p* = 0.011) and 4‐week time points (Paired sample *t*‐test—*p* < 0.001) (Figure 2b).

When comparing the skin sebum results of both clinical assessment (Wilcoxon signed‐rank test—*p* = 0.454 at 2 weeks; Wilcoxon signed‐rank test—*p* = 0.509 at 4 weeks) and sebum content tests (Wilcoxon signed‐rank test—*p* = 0.018 at 2 weeks; Wilcoxon signed‐rank test—*p* = 0.555 at 4 weeks) at 2 and 4 weeks with those at baseline, no significant differences were found (*p* > 0.05) (Figures 1f and 2c).

## 4 Discussion

Acne is a multifactorial inflammatory disease of the pilosebaceous unit. Although benzoyl peroxide, clindamycin, and adapalene are recommended first‐line treatments, their long‐term use can compromise the skin barrier, a factor often implicated in the progression of acne [2, 11, 12, 13, 14]. Consequently, there has been increasing research interest in herbal and botanical extracts, owing to their proposed anti‐inflammatory and antimicrobial properties and their generally favorable tolerability profiles [15].

However, the recently published 2024 American Academy of Dermatology acne management guideline indicates that the current evidence regarding botanical and plant‐derived agents remains insufficient to support definitive recommendations for their efficacy or safety in acne treatment [8].

The present study showed that after 4 weeks of treatment with a herbal balance solution, participants showed a significant reduction in acne grades, closed comedones, and inflammatory papules, suggesting preliminary clinical improvements in patients with mild to moderate acne. The solution contains nine components, including *Houttuynia cordata* and *Glycyrrhiza Glabra* . However, open comedones did not significantly improve, possibly due to the short 4‐week observation period or an inability of the formula to dissolve oxidized sebum plugs.

The pathophysiology of acne involves several factors, but inflammation is a key process [16, 17]. The observed reduction in inflammatory papules may be plausibly attributed to the synergistic anti‐inflammatory properties of its herbal components. Notably, ingredients such as *Houttuynia cordata* , *Saposhnikovia divaricata*, bitter apricot seed extract, and various flavonoids from licorice, citrus peel, and other extracts are known to suppress key inflammatory mediators like interleukins (IL‐1α, IL‐1β, and IL‐6) and tumor necrosis factor‐α (TNF‐α) [18, 19, 20, 21, 22, 23, 24, 25]. By modulating inflammatory pathways, the formulation may have contributed to reductions in erythema and swelling associated with papules, which are consistent with the observed clinical changes [26, 27, 28].

In addition to anti‐inflammatory action, the formula may influence other key acne pathways. Several components, including licorice flavonoids, *Houttuynia cordata* , *Prunus armeniaca* , and *Camellia sinensis* , exhibit antibacterial activity against *Cutibacterium acnes*, a bacterium central to acne‐related inflammation [25, 29, 30, 31, 32, 33]. Furthermore, polyphenols within *Glycyrrhiza glabra* and *Camellia sinensis* may help regulate sebum production [25, 33], another critical factor in acne pathogenesis. The inclusion of lactic acid, a mild alpha‐hydroxy acid, likely contributed to the observed reduction in closed comedones by promoting exfoliation and preventing follicular blockage [34, 35].

A key finding was the significant improvement in skin barrier function, evidenced by increased stratum corneum moisture and decreased transepidermal water loss (TEWL). This is crucial, as a compromised skin barrier can exacerbate acne. These observed changes are likely attributable to ingredients like *Betula alba* extract, known to enhance skin hydration, and lactic acid [34, 36]. Importantly, the solution was well‐tolerated, with no adverse events reported. High safety and minimal irritation are critical for ensuring patient compliance in long‐term acne management.

Although this study was conducted as a prospective, open‐label, single‐arm pre–post evaluation, several measures were implemented to minimize assessment bias. Global acne severity was assessed using an assessor‐blinded photographic evaluation based on standardized facial images, which were anonymized, pooled across time points, and presented in a randomized order to a trained dermatologist blinded to visit sequence and clinical information.

This study used a single‐arm, full‐face application design, a decision made after careful consideration of the scientific, practical, and ethical limitations associated with a split‐face model. A primary scientific concern with a split‐face design is the potential for natural asymmetry in acne presentation. A significant baseline difference in lesion count or severity between the two sides of the face could obscure or confound the true therapeutic effect of the product, thereby impacting the accuracy and reliability of the results. From a practical standpoint, ensuring the precise and consistent application of a liquid formula to only one half of the face is difficult. This challenge is compounded by the risk of inadvertent product crossover, particularly in facial areas prone to acne like the T‐zone, which could contaminate the control side and compromise data integrity. Furthermore, ethical considerations were paramount. Inducing an asymmetrical facial appearance, where one side improves and the other does not, could negatively impact the psychological well‐being and social interactions of a participant. To protect the rights and welfare of participants, the full‐face approach was chosen to avoid this potential burden. This decision was further supported by the fact that key physiological indicators measured in this study, such as comedones and sebum levels, typically do not exhibit significant spontaneous improvement within a short 4‐week period, reducing the immediate need for a concurrent placebo control on the other side of the face for this preliminary exploratory study. This approach aligns with methodological guidelines for single‐arm trials prioritizing ethical feasibility when parallel controls are impractical [37]. We did, however, attempt to mitigate other confounding variables by standardizing skincare routines, advising stable dietary habits, and aligning the 28‐day study period with the menstrual cycles of female participants to minimize hormonal fluctuations [9, 38, 39, 40]. Nonetheless, future studies should incorporate a placebo‐controlled design to provide a higher level of evidence.

Our findings align with recent research exploring botanical formulations for acne. For example, a study by De Lucas et al. also reported that a cream gel with plant‐derived compounds reduced acne lesions over 8 weeks [41]. Although their formulation and study design differed, notably including microbiome analysis, it corroborates the potential of plant‐based actives in acne treatment and highlights an opportunity for future research to investigate the mechanisms of our herbal solution more deeply.

In conclusion, this study provides preliminary evidence that the Herbal Balance Solution was associated with improvements in acne‐related clinical parameters and skin barrier function, with a favorable safety profile, in patients with mild to moderate acne. Its potential as a comprehensive management strategy is promising. However, several limitations must be acknowledged. Although the post hoc analysis indicated high statistical power for the primary endpoint, this study was exploratory in nature and lacked a priori sample size estimation. The relatively small sample size (*n* = 33), short treatment duration, single‐arm open‐label design, and reliance on a single blinded assessor for global severity evaluation warrant cautious interpretation of the findings. Accordingly, future studies should incorporate randomized, assessor‐blinded, placebo‐controlled designs with prospectively powered larger sample sizes and longer follow‐up periods to confirm efficacy, assess durability of response, and further elucidate the active components and mechanisms underlying the observed clinical benefits.

## Author Contributions

Li Li contributed to the conceptualization, methodology, design, and preparation of the manuscript (reviewing and editing). Xi Wang conceptualized this study and contributed to the methodology, design, and writing of the manuscript (reviewing and editing). Xiaohong Shu and Wei Huo contributed to the investigation, formal analysis, and drafting of the original manuscript. Zhaoxia Li and Lin Zou contributed to the investigation and formal analysis. Xiaoyue Teng contributed to the preparation of the original draft.

## Funding

The work was funded by Sichuan Provincial Administration of Traditional Chinese Medicine (2023MS108).

## Ethics Statement

The protocol was approved by the Ethics Committee in 2023 (2023年审(53)号). Informed consent was obtained from all the participants.

## Conflicts of Interest

The authors declare no conflicts of interest.

## Data Availability

The data that support the findings of this study are available from the corresponding author upon reasonable request.
